# Polysaccharides Coatings on Medical-Grade PVC: A Probe into Surface Characteristics and the Extent of Bacterial Adhesion 

**DOI:** 10.3390/molecules15021007

**Published:** 2010-02-23

**Authors:** Ahmad Asadinezhad, Igor Novák, Marián Lehocký, František Bílek, Alenka Vesel, Ita Junkar, Petr Sáha, Anton Popelka

**Affiliations:** 1 Polymer Centre, Faculty of Technology, Tomas Bata University in Zlín, T.G.M Sq. 275, 762 72 Zlín, Czech Republic; 2 Polymer Institute, Slovak Academy of Sciences, Dúbravská cesta 9, 842 36 Bratislava, Slovakia; 3 Tomas Bata University in Zlín, T.G.M. Sq. 5555, 760 01 Zlín, Czech Republic; 4 Plasma Laboratory, Department of Surface Engineering, Jožef Stefan Institute, Jamova cesta 39, SI-1000 Ljubljana, Slovenia

**Keywords:** antibacterial compound, polysaccharide, polyvinyl chloride, chitosan, pectin

## Abstract

Medical-grade polyvinyl chloride was coated by polysaccharides through a novel physicochemical approach. An initial surface activation was performed foremost via diffuse coplanar surface barrier discharge plasma in air at ambient temperature and pressure. Then, radical graft copolymerization of acrylic acid through grafting-from pathway was directed to render a well-defined brush of high density, and finally a chitosan monolayer and chitosan/pectin alternating multilayer were bound onto the functionalized surfaces. Surface characteristics were systematically investigated using several probe techniques. *In vitro* bacterial adhesion and biofilm formation assays indicated that a single chitosan layer was incapable of hindering the adhesion of a *Staphylococcus aureus* bacterial strain, while up to 30% reduction was achieved by the chitosan/pectin layered assembly. On the other hand, chitosan and chitosan/pectin multilayer could retard *Escherichia coli* adhesion by 50% and 20%, respectively. Furthermore, plasma treated and graft copolymerized samples were also found effective to diminish the degree of adherence of *Escherichia coli*.

## 1. Introduction

The topic of biointerface has recently been drawing special attention to this intersection of physics, chemistry, and biology. The motivation for biointerface science studies stems from the urgent need to increase our knowledge on interactions between biomolecules and materials surfaces [1]. Of particular importance is the vulnerability of biomaterial surfaces to contamination with potentially pathogenic bacterial strains through adhesion and biofilm development. This poses a critical challenge for further use of biomaterials on account of the associated infections [2,3]. Hence, controlling the adherence of bacteria onto materials surfaces is highlighted as an important aim in the realm of biointerface science. 

A multistep physicochemical approach making use of plasma technology combined with wet chemistry has fueled considerable interest in delivery of surface-active anti-adherence materials [4]. In the first step of the approach, concerning an inherent lack of suitable functional groups on pristine substrates, plasma treatment at low temperature and atmospheric pressure has been substantiated to be a useful approach yielding reactive entities on the surface [1,5]. Low temperature atmospheric pressure plasmas have been put into the spotlight by virtue of the possibility of rapid, efficient in-line processing without the need for costly vacuum apparatus [6]. However, the need for shorter treatment durations on the order of a few seconds remains a pressing obstacle to extensive applications of this type of plasma [7]. To eliminate the underscored downsides, a novel technology named diffuse coplanar surface barrier discharge (DCSBD) has been devised [8] which enables the generation of a uniform plasma layer under atmospheric pressure with a high surface power density in the very close contact with the treated specimen. DCSBD is based on surface dielectric barrier discharge with a high density of thin discharge channels generated on a dielectric surface in parallel with the sample surface. The plasma is in a good contact with the sample, which appreciably shortens the treatment time and lessens energy consumption [9]. In the second phase of the approach, an end-functionalized polymer brush is synthesized on the surface via surface-initiated polymerization (SIP). SIP encourages the formation of surface-confined thick brush layers of high grafting density on the surface using generation of appropriate initiators anchored to the substrate in which monomers are able to easily make their way through the already-grafted layer and contribute to the chain growth [10,11]. In the final step, biomolecular species are immobilized onto this platform to provide the sought after biological activity. Immobilization of biomolecules onto the surface is feasible in light of established bioconjugation chemistry [12]. 

**Scheme 1:**
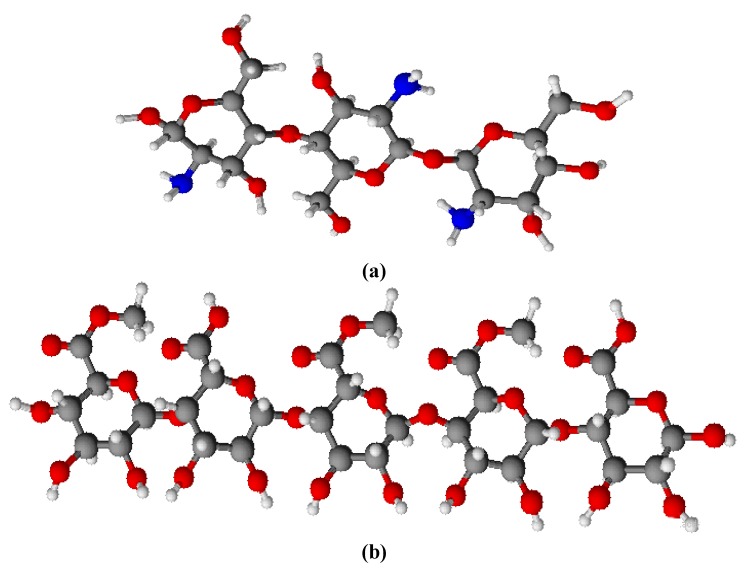
(a) Ball-and-stick models of chitosan and (b) pectin units.

Biocidal biopolymers offer promise for cutting down the environmental complications by diminishing the residual toxicity of the agents, increasing their efficiency and selectivity, as well as protracting biocides lifetime. Polymeric antimicrobial agents also have the advantage of not permeating through skin thanks to their nonvolatility and chemical stability [4]. A typical instance of this class is chitosan (Scheme 1a) which is a cationic polysaccharide derived from deacetylation of chitin and used in pharmaceutical, cosmetic, and food industry applications [13]. It is a linear polysaccharide composed of randomly distributed *β*-(1-4)-linked D-glucosamine and *N*-acetyl-D-glucosamine, which in contact with bacterial cell wall disrupts the cell metabolism and ultimately kills the bacteria. In addition to its direct microbial activity, other studies suggest that chitosan induces a series of defense reactions correlated with enzymatic activities [13,14,15]. Pectin, a structural heteropolysaccharide contained in the primary cell walls of terrestrial plants, is one of the most widely investigated polysaccharides in the field of colon-specific drug delivery. The characteristic structure of pectin is a linear chain of *α*-(1-4)-linked D-galacturonic acid that forms the backbone (Scheme 1b). Pectin has been well-established as an effective gelling and thickening agent, as well as stabilizer for foods [16]. The attraction between polyelectrolytes of opposite charges can create a range of structures including multilayers using layer-by-layer assembly. The prospective applications of biopolyelectrolyte multilayers are mainly in biomedical areas as encapsulation systems, and coatings which can control cell adhesion [17]. It has been shown that chitosan gives stable alternating multilayers with pectin over solid surface and the binding of the biopolymer onto the surface was irreversible over the time scale of the experiments. Indeed, this can be exploited to fabricate analogous multilayer assembly over a polymeric substrate, thereby rendering a higher amount of chitosan onto the surface [14]. 

Numerous works have been dedicated to the study of polysaccharides as antibacterial coatings on polymeric supports [14,15,18,19,20,21,22,23,24,25,26,27,28]. Chen *et al.* [18] immobilized chitosan species via glutaraldehyde onto poly(*N*-isopropylacrylamide)/polypropylene composites using plasma treatment and photo-induced graft polymerization and achieved up to 90% reduction in bacterial colonies of *Staphylococcus aureus* (*S. aureus*) and *Escherichia coli* (*E. coli*) strains. Elsewhere, Yang *et al.* [19] using the same approach, immobilized chitosan units onto poly(ethylene terephthalate) (PET) and obtained effectively antibacterial PET fibers suitable for wound healing purposes. Huh *et al.* [20] coated chitosan entities onto PET textures by plasma glow discharge and acrylic acid (AA) grafting and reported up to 75% bacterial growth inhibition after coating with chitosan. Similar results were reported elsewhere, where atmospheric pressure plasma was employed to deposit chitosan onto PET textiles [21]. Also, PET fibers in another work [15], were treated by *γ*-ray irradiation, grafted with acrylic acid, and further coated by chitosan via esterification, and significant bacterial growth inhibition was finally attained. Similar results were reported by Nigmatullin *et al.* [22] after tethering chitosan onto cellulose membranes, where higher antimicrobial activity was observed for Gram-positive than Gram-negative bacteria. Tseng *et al.* [23] using open air plasma treatment grafted chitosan species onto nylon textiles and obtained significantly improved antibacterial activity. Elsewhere, nonwoven polypropylene (PP) and cotton fabrics were also treated by chitosan which appreciably enhanced antibacterial properties [24]. Hu *et al.* [25] grafted AA to ozone-treated poly (3-hydroxybutyric acid) and poly (3-hydroxy-butyric acid-co-3-hydroxyvaleric acid) membranes and then anchored chitosan entities onto the surface and assessed its biocidal activity against several bacterial strains and reported that *E.coli* was the most susceptible strain, even more than *S. aureus.* In another paper [26], the same authors followed the same strategy for different polyesters and found better antibacterial property against *E. coli* as well. El-tahlawy *et al.* [27] treated cotton fabrics with chitosan in the presence of different crosslinking agents and reported broad-spectrum antibacterial performance against bacteria and fungi. Yang *et al.* [28] treated polysulfone membranes with ozone to introduce peroxides and then grafted AA, followed by coupling of chitosan and reported of a strong biocidal activity against both gram-positive and negative bacteria. Elsabee *et al.* [14] modified PP films by corona discharge and then deposited chitosan and chitosan/pectin multilayer. They reported of a better antibacterial performance for the latter than chitosan monolayer ascribed to higher stability of the multilayer, as it was supported in a thorough study by Marudova *et al.* [17].

**Scheme 2:**
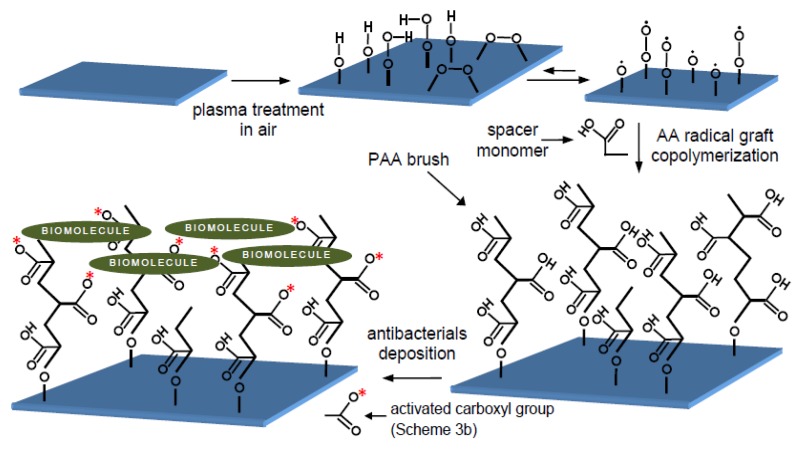
Multistep strategy for biomolecular binding onto PVC substrate.

Despite the outstanding position of polyvinyl chloride (PVC) among medical polymers as well as the importance of chitosan and pectin as marketed biodegradable polysaccharides, to the best of our knowledge no pertinent work has been published hitherto in the literature concerning polysaccharides coating onto PVC films. This attempt is undertaken to contribute to the biointerface discussions surrounding the interactions of medical-grade PVC surface-immobilized polysaccharides with Gram-positive and Gram-negative bacterial strains. This is accomplished through bringing the binding of chitosan monolayer and chitosan/pectin multilayer via the aforesaid multistep physicochemical approach (Scheme 2) into special focus. Surface characteristics and bacterial adhesion extent are then investigated by the relevant probe methodologies.

## 2. Results and Discussion

### 2.1. Surface wettability

A highly surface sensitive technique is contact angle analysis which enables a convenient assessment of the surface wettability. Table 1 includes the contact angle values of deionized water (*θ_w_*) recorded on different samples. Each sample has been designated by a number from 1 to 5 whose notation is inserted in the title of Table 1. Based on the given data, Sample 1 exhibits a hydrophobic characteristic which after being treated by plasma, an evident change in *θ_w_* arises and hydrophilicity ascends as anticipated. This trend continues as to Sample 3 on which polyacrylic acid (PAA) chains are grafted where more hydrophilic propensity is shown inferred from *θ_w_* value. The elevated hydrophilicity upon multistep modifications is assumed to come from the inclusion of superficial hydrophilic entities [31,32]. The hydrophilicity then decreases as polysaccharides are coated onto the surface, though is well higher than that of Sample 1, as the inherent hydrophilicity of chitosan is beyond doubt [21,24,28]. Furthermore, Sample 5 exhibits higher wettability than Sample 4 implying a more effective binding of chitosan onto the surface, as remarked in other efforts as well [14]. To further explore the physicochemical parameters of the examined surfaces, an extensively used theory, Lifshitz-van der Waals/acid-base (LW/AB) [33], has been exploited for free surface energy evaluation whose outputs with reference to diiodomethane, ethylene glycol, and deionized water as wetting liquids are supplied in Table 1. Sample 1 exhibits a basic character (*γ^-^*>*γ^+^*) as proposed by the data, even though acidity or basicity of neat PVC is yet controversial [2,33,34]. Subsequent to the plasma treatment, the total surface free energy (*γ^tot^*) is raised in terms of the contact angle values. This increase is principally assisted by the polar (acid-base) component (*γ^AB^*), rather than the apolar one (*γ^LW^*), implying an incorporation of superficial polar oxygen-containing entities thanks to the air plasma treatment. A significant rise in *γ^tot^* and *γ^AB^* values is noticed for Sample 3, in comparison with Samples 1 and 2, indicative of the presence of carboxyl-containing units on the surface. As for Samples 4 and 5, a reduction in *γ^AB^* and *γ^tot^* values is observed compared to Sample 3, however, their *γ^tot^* values rise above that of Sample 1. The minimum values of *θ_E_* and *θ_F_* are found for Sample 5 which reflect that the surface is seemingly coated by alcoholic and amine containing moieties which in fact points to the more efficient binding of chitosan when compared to Sample 4. It is then speculated that pectin favors surface attachment of chitosan entities also been remarked in the literature [14,17].

**Table 1:** Contact angle analysis results of different specimens using deionized water (w), ethylene glycol (E), diiodomethane (D), and formamide (F) as wetting agents. Sample 1: pristine/control; Sample 2: plasma treated: Sample 3: PAA grafted; Sample 4: chitosan coated; Sample 5: chitosan/pectin coated (mean± standard deviation).

Specimen	*θ_w _(˚)*	*θ_E _(˚)*	*θ_D _(˚)*	*θ_F _(˚)*	*γ^-^*_LW/AB_	*γ^+^*_LW/AB_	*γ^AB^*_LW/AB_	*γ^LW^*_LW/AB_	*γ^tot^*_LW/AB_	*γ* ^a)^_Wu_	*γ* ^b) ^_KN_	*γ* ^c) ^_LN_
					(mJ/m^2^)	(mJ/m^2^)	(mJ/m^2^)	(mJ/m^2^)	(mJ/m^2^)	(mJ/m^2^)	(mJ/m^2^)	(mJ/m^2^)
Sample 1	85.9	60.5	43.5	64.2	5.1	0.0	1.0	37.8	38.8	37.8	33.3	33.6
	(±2.5)	(±3.0)	(±3.5)	(±6.0)								
Sample 2	64.9	49.4	36.2	51.0	24.9	0.5	6.7	41.5	48.2	41.5	40.4	40.7
	(±3.0)	(±4.0)	(±5.5)	(±6.0)								
Sample 3	46.5	51.3	38.0	47.7	62.9	2.7	26.1	40.6	66.7	51.9	43.1	43.4
	(±4.0)	(±5.5)	(±5.0)	(±4.5)								
Sample 4	63.7	43.4	28.2	44.9	22.2	0.3	4.9	45.0	49.9	45.0	42.8	43.0
	(±5.5)	(±3.0)	(±2.5)	(±5.0)								
Sample 5	50.5	40.0	31.5	31.0	42.2	0.6	10.5	43.6	54.1	50.0	46.4	46.6
	(±3.5)	(±2.5)	(±4.5)	(±3.5)								

^a) ^Surface free energy value according to Wu equation of state [33]; ^b) ^Surface free energy value according to Kwok-Neumann model [33]; ^c)^Surface free energy value according to Li-Neumann model [33].

To make an analogy among LW/AB theory and equation of state models, the predictions for surface free energy values of Samples 1-5 based on the three equation of state models [33] (Kwok-Neumann, Li-Neumann, and Wu) using four wetting agents are also included in Table 1. Although, they produce lower outputs compared to LW/AB approach, the variation trend from Sample 1 to 5 sustains. The Wu equation of state yields closer data to LW/AB predicted ones on the grounds of the respective underlying assumptions.

**Figure 1:**
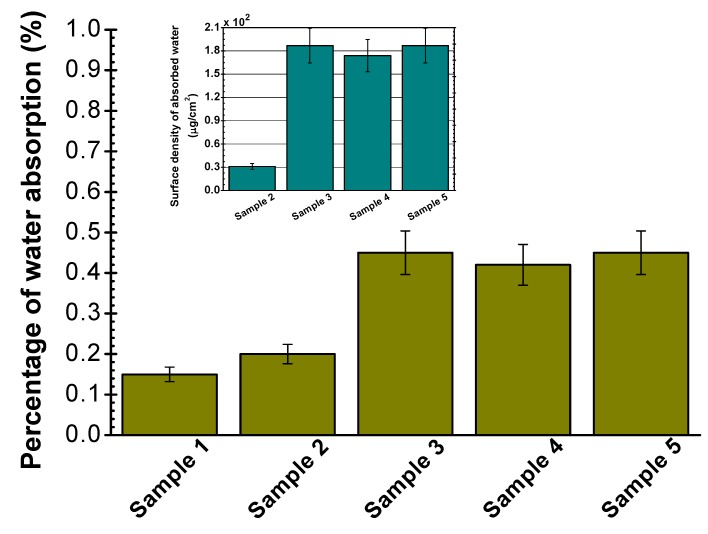
Percentage of water absorption after 24 h for Samples 1-5. The inset shows the corresponding surface density of absorbed water (error bars depict standard deviations).

The water absorption test quantities, given as histograms in Figure 1, back up the contact angle results. It is learned that the water absorption capacity is boosted by an improvement in the surface free energy as verified in other reports [14,35,36,37]. In point of fact, the hydrophilic modification gives an impression of the capacity for water absorption. The surface modified specimens (Samples 2–5) turn out to be more hygroscopic compared to pristine sample which shows a minimal uptake. Figure 1 also embraces an inset where the corresponding surface densities of the absorbed water (absorbed water mass/surface area subtracted from pristine sample) are demonstrated. Surface density of the absorbed water can be utilized as a benchmark to score some insight into the extent of the applied modifications, since they correlate with the surface free energy values.

### 2.2. Surface morphology

**Figure 2:**
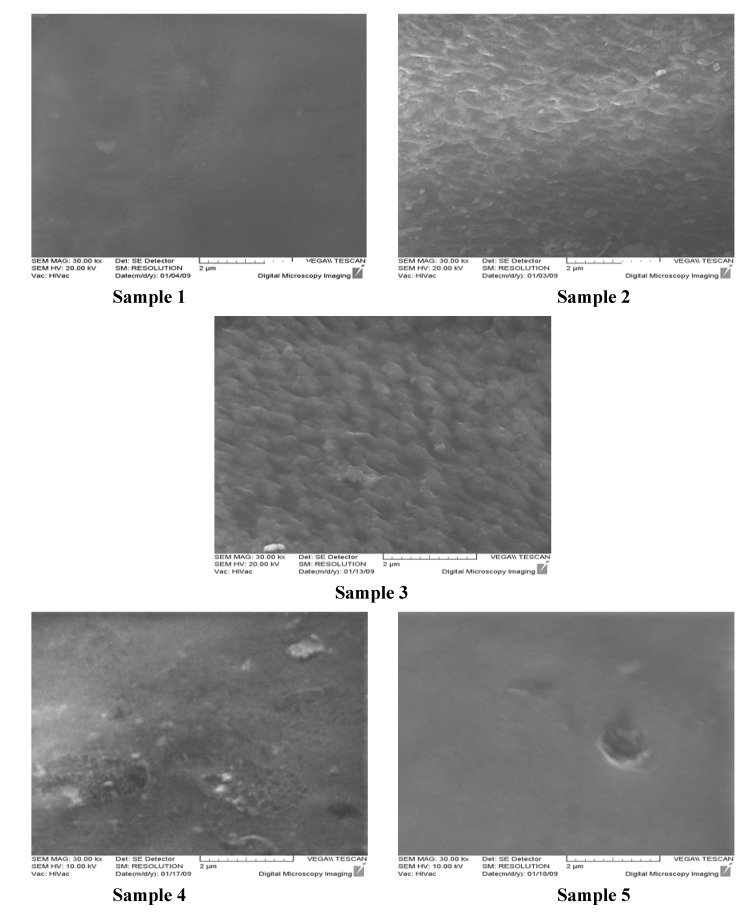
SEM micrographs of Samples 1-5 taken at 30 000 × magnification.

The surface topography of Samples 1-5 investigated by SEM as a common surface qualitative technique are presented in Figure 2. Sample 1 shows a level and uniform morphology which goes through a significant alteration during the plasma treatment taking on an etched pattern with an unevenly shaped texture. The morphology generated is favorable for the subsequent coupling processes due to an enhanced surface area and roughness [6]. The developed pattern on Sample 2 is indeed, an outcome of the competing functionalization and ablation phenomena which brings on a reorganization of the surface microstructure [38].

The incident of the ablation is validated by gravimetric analysis where a weight loss of 4 μg·cm^-2^ has been observed due to the plasma treatment for 15 sec implying an approximate etching rate of 2 nm/s in terms of the used PVC grade density. Based on the micrograph of Sample 3, PAA chains develop superficial domains of submicron dimension and brush-like features are then recognizable on the surface. As the grafting moves forward, clustering takes place because of the domains size growth. An additional compelling factor in controlling the surface microstructure is the grafting mechanism which is actually initiated by generated surface radicals. A portion of the generated radicals may also be present in sublayer and begin the grafting reaction. This brings forth evidently bulged grafted top layer following the diffusion of monomers and participation in chain propagation [39]. In fact, Sample 3 exhibits the roughest topography compared to the others, where a brush-like pattern comes into view which renders an active platform for subsequent modification. A similar finding was also reported on AA-grafted polyurethane [40]. Assuming an evenly distributed polymer brush, the average graft density of PAA is gravimetrically estimated to be ≈185 μg·cm^-2^ which is higher than the mean values reported in similar works focused on surface grafting of PAA [28,39,41]. It is interesting to note that the estimated graft density falls on the order of the corresponding surface absorbed water density calculated in the previous section. Moreover, the grafted brush dry thickness should be around 1.5 μm with respect to the approximate PAA homopolymer density (1.1 g·cm^-3^[39]). Chitosan coating as well as chitosan/pectin multilayer alter the surface topography, as observed in Figure 2, leading to relatively even and more uniform textures, in particular for Sample 5. It is postulated that PAA chains on the surface are pushed apart in order to accommodate bulky polysaccharide species reflected in a filling up action which brings about a comparatively leveled morphology [39]. Morphological findings suggest that chitosan is more effectively bound onto the surface when it forms a layered assembly with pectin. This is in agreement to what commented in the former section regarding the beneficial role of pectin in quality of the chitosan attachment onto the surface.

### 2.3. Surface chemistry

**Figure 3:**
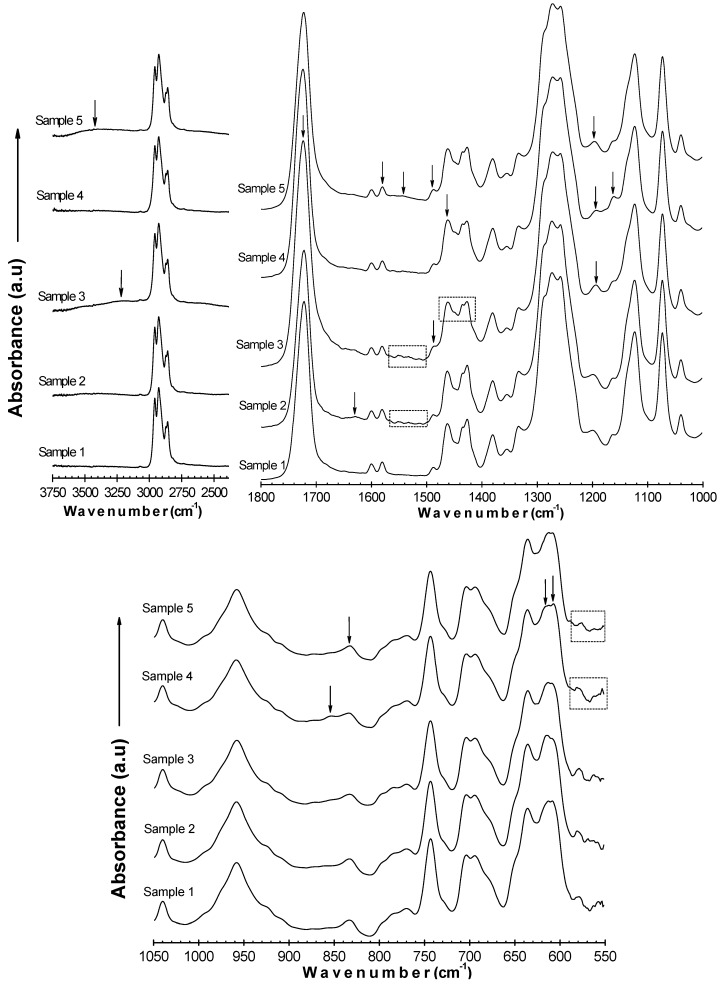
ATR-FTIR spectra of Samples 1–5 split over three wavenumber ranges.

ATR-FTIR is extensively utilized as a surface analysis technique to provide semiquantitative information on the chemistry of the near-surface region. However, as reported in the literature [42], the mean probe depth of ATR-FTIR equipped with ZnSe crystal for a PVC of refractive index measuring 1.5 lies on the order of 4 μm which goes beyond the regular thickness of modified layers on a substrate. Figure 3 displays the infrared spectra of Samples 1-5 separated over three wavenumber ranges for clarity purpose. In the spectrum of Sample 1, one can readily identify the characteristic bands of ester, and carbonyl-containing groups, besides neat PVC typical signals. This manifests the existence of some additives in the current medical-grade PVC. After exposing the samples to plasma, no pronounced change is detected regarding the Sample 2 spectrum, compared to Sample 1. This is because of signals overlapping leading to masking the plasma effects, and also due to the limited depth of plasma modification to outermost layers which thus, can not be well explored by ATR-FTIR. In other words, the top nanoscale superficial change is such diluted that the information is virtually lost. Nevertheless, a new absorption of weak strength comes into view around 1630 cm^-1^ which relates to C=C stretching vibration as a result of the dehydrochlorination phenomenon [43]. 

According to the spectrum of Sample 3, a broad and weak peak appears at 3,000–3,400 cm^-1^ which is assigned to the H-bonded –OH stretching vibration in carboxylic acids. Also, C–H stretching bands within the 2,800–3,000 cm^-1^ range gain intensity. The typical C=O stretching mode at 1,720 cm^-1^ is amplified. The signals appearing within 1,400–1,500 cm^-1^, corresponding to CH_2_ vibration, CH_3_ deformation, and –OH bending modes, marginally gain strength, as do the signals within the 1,100–1,300 cm^-1^ range related to C–O stretching vibrations in carboxylic compounds, as well as CH_2_ bending mode. The peaks appearing within the 600-700 cm^-1^ range connected with C–Cl stretching decrease in height. These alterations bear out that PAA units are coupled onto the surface. As to the spectrum of Sample 4, due to the coincidence of signals, no significant changes are noted. The C=O stretching peak at 1,720 cm^-1^ loses strength. The intensity of the peak around 1460 cm^-1^, likely due to C–N stretching vibration, slightly increases. The signal at 1,190 cm^-1^ corresponding to C–O–C stretch in esters abates, while the absorption at 1,160 cm^-1^ associated with C–O–C in cyclic ethers and C–C–N bending in amines gains strength. A minor, broad peak appears around 850 cm^-1^, assigned to NH_2_ wagging in amines. The signal at 605 cm^-1^ intensifies, which can be related to–OH deformation in phenols, while the signals appearing around 612, 575, and 560 cm^-1^ and which correspond to O–C–O bending in esters and O–C=O bending in carboxylic acids decrease in magnitude. It is concluded from the aforementioned changes that chitosan species are present on the surface. Chitosan/pectin coating leads to some relevant changes in the examined surface chemistry. A broad peak emerges within the 3,200–3,600 cm^-1^ range characteristic of –OH and –NH stretch. The C=O stretching peak at 1,720 cm^-1^ loses strength. A weak, broad signal appears around 1,540 cm^-1^, and also the intensity of the 1,580 and 1,485 cm^-1^ absorptions, which all may be associated with –NH deformation, increase slightly. A signal around 830 cm^-1^ gains strength, likely due to NH_2_ wagging in amines. Furthermore, the signals appearing around 575 and 560 cm^-1^, which correspond to O–C–O bending in esters and O–C=O bending in carboxylic acids, decrease in magnitude. Hence, ATR-FTIR lends support to the presence of chitosan/pectin assemblies onto the surface.

**Figure 4:**
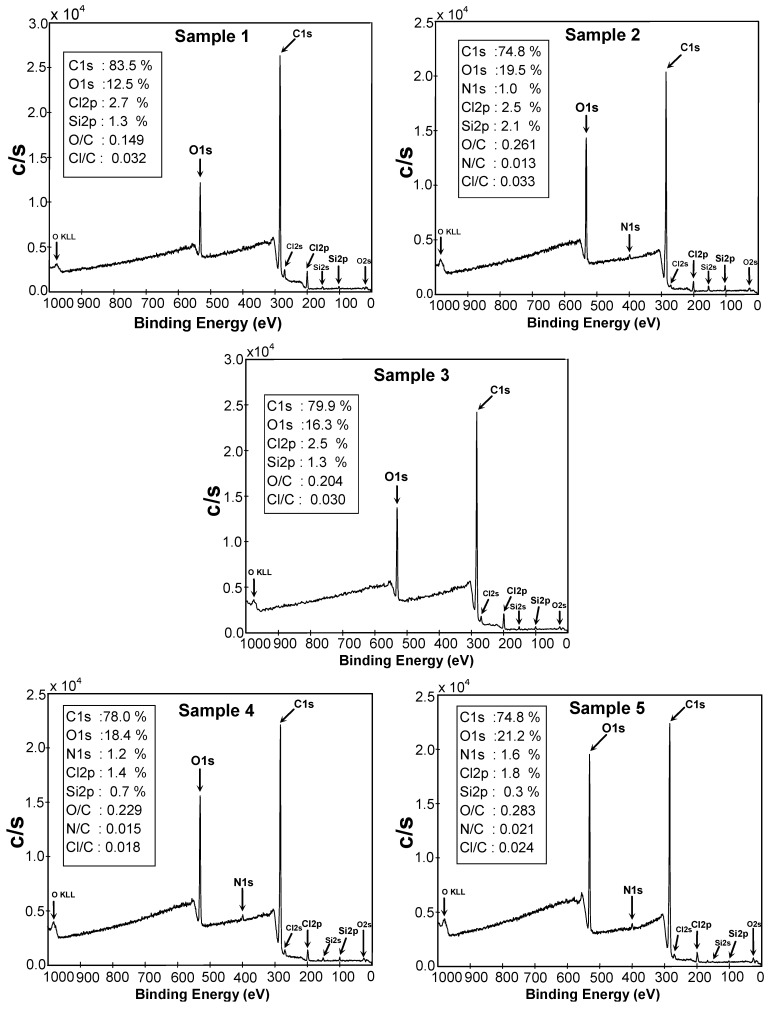
XPS survey-scan spectra of Samples 1-5 along with atomic compositions.

XPS, with a probe depth measuring around 5 nm [44], has been put to use to more thoroughly monitor the bearings of the surface modifications by picking up a quantitative perception into the surface elemental composition. The recorded survey spectra along with the corresponding surface atomic compositions and ratios of Samples 1-5 are all provided in Figure 4. The elements carbon (C), oxygen (O), chlorine (Cl), and silicon (Si) are found on the surface of Sample 1, whose composition and elemental ratios are presented in the legend of the respective graph. The Cl2p atomic content is substantially lower than the amount found for neat PVC containing no additives, which refers to the presence of several additives and also X-ray degradation [45]. The same rationale accounts for the considerable amount of O1s, which is not a typical element in standard PVC, detected in Sample 1. XPS also recognizes some trivial quantity of silicon which may be associated with the production/molding process as a contaminant. An ostensible change in C1s, O1s, and Cl2p core-level signals intensity arises for Sample 2 quantitatively expressed in the legend of the graph. It is easily seen that the Cl2p intensity decreases while that of O1s noticeably increases, which alludes to the introduction of superficial oxygenated entities together with dehydrochlorination as a result of the ablation. Nitrogen (N1s) is also found on the surface because the plasma treatment has been implemented in air medium. The O/C ratio increases while Cl/C one declines when compared to those of Sample 1. The amount of Si2p on the Sample 2 surface increases supposedly coming from plasma parts as a pollutant.

Subsequent to the PAA grafting, the O1s atomic content decreases compared to Sample 2; however, it increases in comparison with Sample 1, although a higher O1s content than in a plasma treated one was anticipated for Sample 3, since a pure PAA layer would deliver an oxygen atomic concentration of around 40%, in accord with the literature [39]. This implies that the detected PAA layer is not pure and PAA chains spread not only in the topmost layer but also in the subsurface layer far beneath the XPS probe depth [39]. The Cl2p content undergoes no change upon PAA grafting and the N1s signal is also no longer seen in Sample 3. The Si2p signal due to the surface coverage attenuates as well. Upon binding chitosan on the surface (Sample 4), pronounced changes appear in the surface chemistry, as O1s content and the O/C fraction increase and the N1s signal also emerges, while the Cl2p and Si2p bands diminish due to the surface coverage by polysaccharide species. This trend continues for Sample 5 as higher O1s and N1s as well as O/C and N/C atomic rations are detectable, compared to Sample 4 giving support to the notion that chitosan can be more stably attached, *i.e.* in higher quantity, onto the surface when layered along with pectin. In other words, the use of pectin can promote the quality of chitosan binding.

### 2.4. Bacterial adhesion and biofilm assay

**Figure 5:**
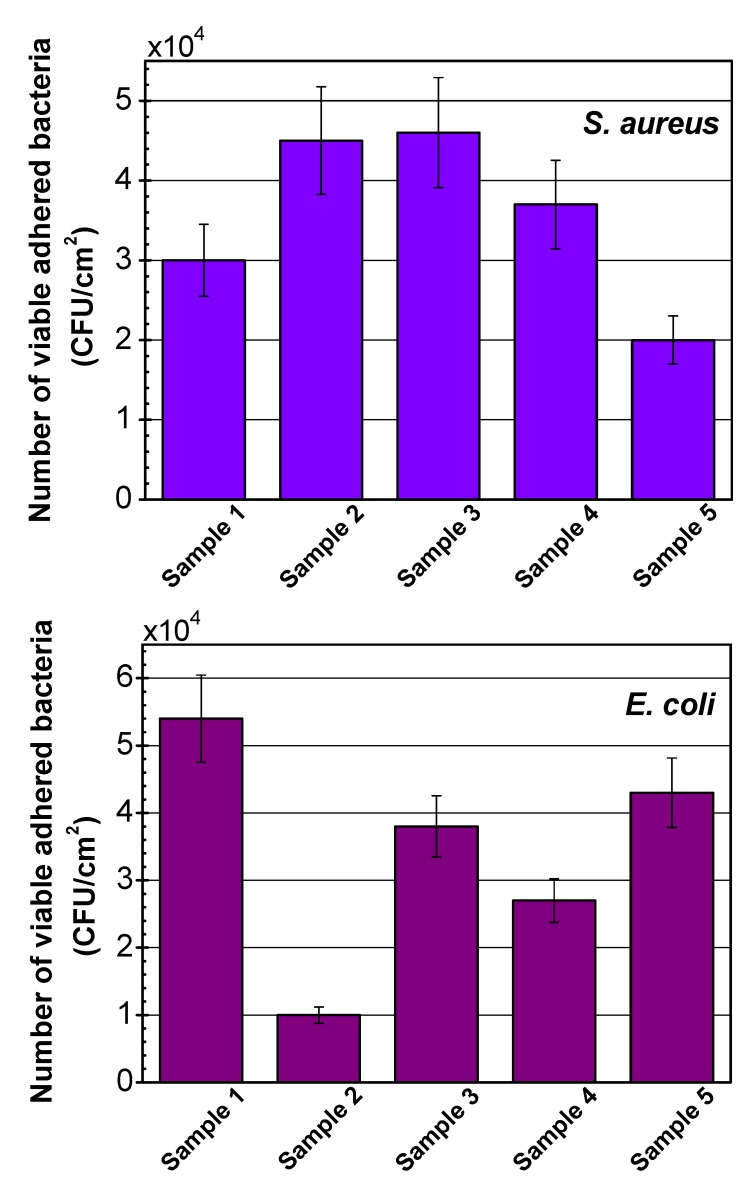
Histograms of bacterial adhesion degree for Samples 1-5 after 24 h incubation against two microorganisms, (error bars depict standard deviations).

The most crucial step in biofilm formation is bacterial adhesion, considered a sophisticated topic in biointerface science, many of whose aspects are not yet been well understood. As a matter of fact, adhesion phenomenon is an interplay of myriad factors [46]. On one hand, bacterial traits, such as cell wall elements, hydrophobicity, and surface charge, play critical roles; while on the other hand, biomaterial surface properties like chemical composition, wettability, roughness, and morphology can significantly influence the adherence. Figure 5 shows the histograms of the extent of bacterial adhesion for Samples 1-5 after 24 h incubation. The results have been evaluated after 24 h incubation in order to provide a better assessment of the biofilm formation. As regards to the degree of adherence of *S. aureus* onto Samples 2-4, no reduction is evident in the number of viable adhered colonies, compared to Sample 1, signifying an inability of the modifications to hamper the adhesion of *S. aureus* to the surface. However, when it comes to Sample 5, 30% inhibition is observed, suggesting the capability of chitosan/pectin to reduce the degree of adherence of the Gram-positive strain. Surface free energy (wettability) and topography appear to be two pivotal elements in regulating the observed degree of adhesion. Indeed, the degree of adhesion correlates with the hydrophilicity and roughness of the samples. From Sample 1 to 3, both hydrophilicity and roughness rise, as remarked earlier, and then decrease in the case of Samples 4 and 5. The adhesion degrees vary with a similar trend as well. Considering Sample 5, it is inferred that chitosan/pectin assembly imparts biocidal effects against *S. aureus.* A distinctive mode is observed as for the *E. coli* adhesion, where Samples 2-5 outdo Sample 1 in the prevention of bacterial adhesion. Plasma treatment hampers the bacterial adhesion by 80% while graft copolymerization of PAA does it by 30% extent. Chitosan single layer and chitosan/pectin multilayer diminish the adherence degree by 50% and 20%, respectively. Chitosan/pectin multilayer is found to be effective against both Gram-positive and Gram-negative strains, which can be interpreted as due to a higher quality of the chitosan coating when it is applied along with pectin. Different mode of action of the tested bacteria on the samples expresses the complex surface structure and motility of *E. coli* compared to *S. aureus*, as outlined in detail elsewhere [47]. The number of *E. coli* adhered onto Sample 1 is higher than that of adhered *S. aureus*, which is ascribed to the different physicochemical attributes of the bacteria and materials [46]. It is already established that Gram-negative *E. coli* possesses flagella on the structures exterior to the cell wall and is mobile. Additionally, *E. coli* has fimbriae, which make the bacterium more adsorbable. This is in agreement with the literature where higher threat of *E. coli* compared to *S. aureus* has been reported [25,26]. Therefore, the multistep modifications are more detrimental to Gram-negative than Gram-positive adhesion. Nonetheless, the antibacterial samples do not fully repress the biofilm formation after 24 h incubation in high cell concentration suspensions, which is in accord with a report from Zhang *et al.* [48] on a different system. Additional efforts in this area will be essential to elucidate the interactions of living microorganisms and biomaterials’ surfaces and their pertinence to physicochemical characteristics.

## 3. Experimental

### 3.1. General

PVC pellets, extrusion medical-grade RB1/T3M of 1.25 g·cm^-3^ density, were obtained from ModenPlast (Italy) and used as received. Pectin from apple (BioChemika, with 70-75% esterification), acrylic acid (99.0%, anhydrous), and *N*-(3-dimethylaminopropyl)-*N′*-ethylcarbodiimide hydrochloride (EDAC, 98.0%) were supplied by Fluka (USA). Chitosan from crab shells with medium molecular weight and a 75-85% degree of deacetylation, sodium metabisulfite (99.0%, Reagentplus), glutaraldehyde (as 25.0 wt% aq. solution), ethylene glycol, (99.8 %, anhydrous), diiodomethane (99.0 %, Reagentplus), formamide (99.5%, molecular biology grade), and Triton X-100 (laboratory grade) were all purchased from Sigma-Aldrich (USA).

### 3.2. Preparation of substrates

PVC granules were molded by hot pressing at 165 ºC for 10 min, formed into flat sheets of 1 mm thickness, and subsequently cut into 4 × 5 cm pieces after cooling. The substrates were then washed thoroughly by rinsing with dilute ethanol, sonicating with 0.1 vol. % aq. solution of non-ionic surfactant (Triton X-100) and deionized water for 10 min at 30 ºC. Finally, they were dried in an air-circulating oven at 30 °C for 24 h.

### 3.3. Plasma activation

**Scheme 3:**
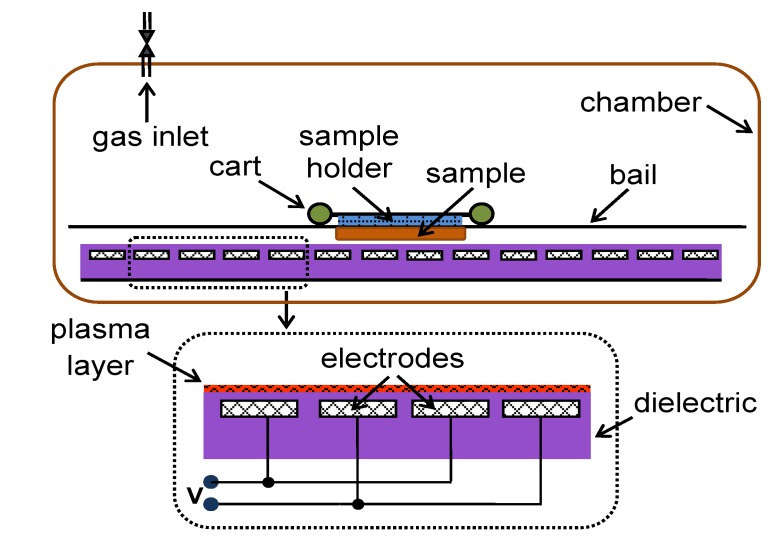
Schematic profile of DCSBD plasma system.

Plasma treatment was implemented under static conditions by the DCSBD technology on a laboratory scale with air as the gaseous medium at atmospheric pressure and room temperature. A schematic profile of the plasma system is given in Scheme 3. It basically comprises a series of parallel metallic electrodes inset inside a ceramic dielectric located in a glass chamber which allows the carrier gases to flow. All samples were treated on both sides with plasma power of 200 W for 15 sec.

### 3.4. PAA grafting

PVC substrates, upon treatment with plasma, were immersed into spacer solutions containing 10 vol. % AA aq. solution. To control radical graft copolymerization of AA onto PVC following surface-initiated pathway (Scheme 2), 0.1 wt. % sodium metabisulfite was added as an efficient reductant to inhibit AA homopolymerization. The reaction was allowed to proceed for 24 h at 30 ºC. The samples were taken out, washed with 0.05 vol.% Triton X-100 aq. solution, and also deionized water for 5 min at 30 ºC in an ultrasonic bath to remove any unbound PAA species on the surface. Drying was done at 30 ºC in an air-circulating oven for 24 h.

### 3.5. Polysaccharides immobilization

**Scheme 4:**
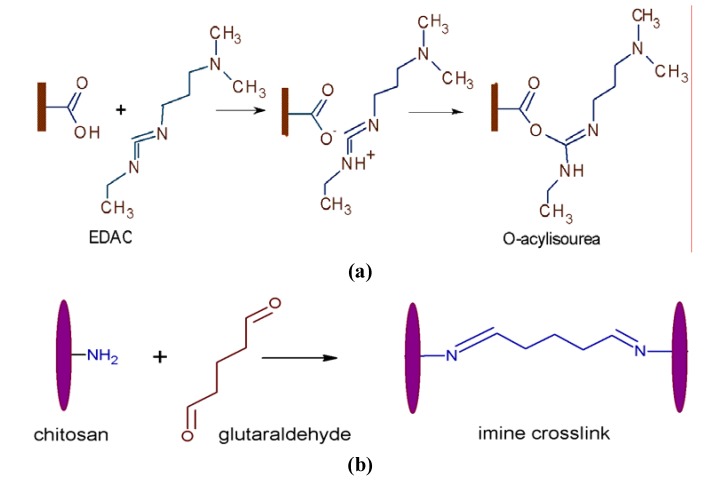
(a) Carboxyl group activation by EDAC and (b) imine crosslink formation following chitosan and glutaraldehyde reaction.

PAA grafted PVC samples were immersed into 0.1 w/v% EDAC aq. solution at 4 ºC for 6 h in order to activate the carboxyl groups on the surface. The activation mechanism is given in Scheme 4a where the highly active key intermediate, O-acylisourea, is produced having potential to react with reducing agents [29]. Subsequently, they were transferred to chitosan solutions prepared as 1 w/v % chitosan in 2 v/v % aq. solutions and kept there for 24 h at 30 ºC. Then, the samples were rinsed several times with deionized water and dipped overnight into 1 w/v % glutaraldehyde aq. solution at 4 ºC which acted as an amine-reactive homobifunctional fixative to immobilize chitosan species onto the surface via crosslinking. Crosslinking takes place by imine formation as a result of a reaction between primary amine and aldehyde [30], as depicted in Scheme 4b. To explore the effect of pectin as a thickener on chitosan layer, PAA grafted samples after being activated by EDAC were dipped into chitosan solution of the concentration stated above for 20 min at room temperature, then withdrawn and dried in air for a few minutes and immediately were transferred into pectin solution (prepared similar to chitosan solution) and kept there for 20 min. This procedure was repeated nine times to deliver chitosan/pectin alternating multilayered coatings on the surfaces. Then, the samples were rinsed several times with deionized water and incubated in 1 w/v % glutaraldehyde aq. solution at 4 ºC overnight. All of samples were fully washed and dried under the same conditions mentioned previously. 

### 3.6. Surface wettability assessment

The wettability of the samples was evaluated using contact angle analysis and water absorption test. Static contact angle measurements by the sessile drop method and the respective data analysis were carried out via Surface Energy Evaluation (See) System (Advex Instruments) equipped with a CCD camera using a set of standard testing liquids at 22 °C and 60% relative humidity. Each resulting contact angle was an average of 10 measured values recorded 30 sec after reposing each drop of 5 μL volume on the sample surface. Water absorption test was implemented by exposing each specimen to water for 24 h at 22 °C and gently padding with a filter paper to eliminate unabsorbed water from the surface. The results were reported as percentage of water absorption [(wet weight - dry weight)/ dry weight] × 100) after taking the mean of three replicates.

### 3.7. Surface topography analysis

Scanning electron microscopy (SEM) was carried out on a VEGA II LMU instrument (TESCAN) operating in the high vacuum/secondary electron imaging mode at an accelerating voltage of 5-20 kV. The samples were sputter coated with a thin layer of palladium/gold alloy and tilted 30˚ to reach an enhanced resolution and stereoscopic view. The images were all taken at 30 000 × magnification.

### 3.8. Surface chemistry probe

ATR-FTIR spectra were collected at a spectral resolution of 2 cm^-1^ via an Avatar 320 FT-IR spectrometer (Nicolet) equipped with a ZnSe crystal at an incident angle of 45º. Each spectrum represents 64 co-added scans rationed against a reference spectrum obtained by recording 64 co-added scans of an empty ATR cell. The acquired spectra were analyzed using OMNIC Software Suite. X-ray Photoelectron Spectroscopy (XPS) was conducted using TFA XPS Physical Electronics. The base pressure in the XPS analysis chamber was ≈ 6×10^-8^ Pa. The samples were excited by X-rays over a 400-μm diameter spot area with monochromatic Al Kα1,2 radiation at 1486.6 eV. The emitted photoelectrons were detected by a hemispherical analyzer positioned at a take-off angle of 45º. Survey-scan spectra were obtained at a pass energy of 187.85 eV and 0.4 eV step resolution. An electron gun was employed for surface neutralization. The elemental concentration analysis was performed over three different positions by MultiPak v7.3.1 software.

### 3.9. In vitro bacterial adhesion assay

Bacterial adhesion and biofilm formation experiments were performed using Gram-positive (*S. aureus* 3953) and Gram-negative (*E. coli* 3954) bacteria. The circular shape specimens (d ≈ 8mm) were cut from the pristine and modified PVC samples before further investigation. The bacterial adhesion was performed as follows, the test tubes with 10 mL of sterile water solution of nutrient broth (Envitech, Czech Republic) was inoculated with given bacterial strain to reach ≈10^8^ CFU·mL^-1^ and left at room temperature for 30 min. Then, the specimens were inserted into the test tubes. After 24 hours incubation at 37 ºC under continuous shaking at 100 rpm, the test tubes were opened and the specimens were carefully removed form the medium, rinsed with sterile distilled water to remove loosely adhered bacteria and placed into other test tubes containing 2 mL of sterile deionized water. The bacteria adhered on the surface of the specimens were removed by vigorous shaking of the test tube at 2000 rpm for 30 sec and quantified by serial dilutions and spread plate technique. A 1 mL aliquot of the suspension was diluted decimally and from each dilution, 0.1 mL was transferred to nutrient agar plate and the surviving bacteria were enumerated after 24 hours cultivation at 37 ºC reported as CFU·cm^-2^. Each experiment was reiterated in triplicate.

## 4. Conclusions

This work highlights the functionality of the adopted multistep physicochemical approach to bind polysaccharide species onto the medical-grade PVC surface. DCSBD plasma is capable of increasing roughness, surface free energy, and introducing oxygen-containing functionalities anchored onto the surface. A structured PAA brush of high graft density is synthesized using surface-initiated approach to further improve hydrophilicity and develop a stable brush-like assembly to yield a platform for biomolecular binding. Surface-sensitive analyses evidence the presence of chitosan and chitosan/pectin multilayer. In vitro bacterial adhesion and biofilm formation assays indicate incapability of single chitosan layer in hindering the adhesion of *S. aureus* bacterial strain, while up to 30% reduction is achieved by chitosan/pectin layered assembly. On the other hand, chitosan and chitosan/pectin multilayer could retard *E. coli* adhesion by 50% and 20%, respectively. Furthermore, plasma treated and graft copolymerized samples are also found effective to diminish the adherence degree of *E. coli*. The findings of this work are expected to shed some light on the issues involved in the biointerface science of polysaccharides coatings on polymeric substrates. 

## References

[1] Desmet T., Morent R., Geyter N.D., Leys C., Schacht E., Dubruel P. (2009). Nonthermal plasma technology as a versatile strategy for polymeric biomaterials surface modification: A review. Biomacromolecules.

[2] Speranza G., Gottardi G., Pederzolli C., Lunelli L., Canteri R., Pasquardini L., Carli E., Lui A., Maniglio D., Brugnara M., Anderle M. (2004). Role of chemical interactions in bacterial adhesion to polymer surfaces. Biomaterials.

[3] Triandafillu K., Balazs D.J., Aronsson B.O., Descouts P., Quo P.T., van Delden C., Mathieu H.J., Harms H. (2003). Adhesion of Pseudomonas aeruginosa strains to untreated and oxygen-plasma treated poly(vinyl chloride) (PVC) from endotracheal intubation devices. Biomaterials.

[4] Kenawy E.R., Worley S.D., Broughton R. (2007). The chemistry and applications of antimicrobial polymers: A state-of-the-art review. Biomacromolecules.

[5] Denes F.S., Manolache S. (2004). Macromolecular plasma-chemistry: An emerging field of polymer science. Prog. Polym. Sci..

[6] Chu P.K., Chen J.Y., Wang L. (2002). Plasma-surface modification of biomaterials. Mat. Sci. Eng..

[7] černák M., černáková L., Hudec I., Kováčik D., Zahoranová A. (2009). Diffuse coplanar surface barrier discharge and its applications for in-line processing of low-added-value materials. Eur. Phys. J. Appl. Phys..

[8] Šimor M., Ráhel J., Vojtek P., černák M. (2002). Atmospheric-pressure diffuse coplanar surface discharge for surface treatments. Appl. Phys. Lett..

[9] Černák M., Ráhel J., Kováčik D., Šimor M., Brablec A., Slavíček P. (2004). Generation of thin surface plasma layers for atmospheric-pressure surface treatments. Contrib. Plasma Phys..

[10] Bhattacharyaa A., Misra B.N. (2004). Grafting: A versatile means to modify polymers techniques, factors and applications. Prog. Polym. Sci..

[11] Zhao B., Brittain W.J. (2000). Polymer brushes: Surface-immobilized macromolecules. Prog. Polym. Sci..

[12] Tripathi S., Mehrotra G.K., Dutta P.K. (2008). Chitosan based antimicrobial films for food packaging applications. e-Polymers.

[13] Rabea E.I., Badawy M.E.T., Stevens C.V., Smagghe G., Steurbaut W. (2003). Chitosan as antimicrobial agent: Applications and mode of action. Biomacromolecules.

[14] Elsabee M.Z., Entsar S.A., Khaled S.A. (2008). Surface modification of polypropylene films by chitosan and chitosan/pectin multilayer. Carbohyd. Polym..

[15] Jou C.H., Lee J.S., Chou W.L., Yu D.G., Yang M.C. (2005). Effect of immobilization with chondroitin-6-sulfate and grafting with chitosan on fibroblast and antibacterial activity of polyester fibers. Polym. Adv. Technol..

[16] Thakur B.R., Singh R.K., Handa A.K. (1997). Chemistry and uses of pectin-A review. Crit. Rev. Food Sci..

[17] Marudova M., Lang S., Brownsey G.J., Ring S.G. (2005). Pectin-chitosan multilayer formation. Carbohyd. Res..

[18] Chen K.S., Ku Y.A., Lee C.H., Lin H.R., Lin F.H., Chen T.M. (2005). Immobilization of chitosan gel with cross-linking reagent on PNIPAAm gel/PP nonwoven composites surface. Mater. Sci. Eng..

[19] Yang M.R., Chen K.S., Tsai J.C., Tseng C.C., Lin S.F. (2002). The antibacterial activities of hydrophilic-modified nonwoven PET. Mat. Sci. Eng..

[20] Huh M.W., Kang I.K., Lee D.H., Kim W.S., Lee D.H., Park L.S., Min K.E., Seo K.H. (2001). Surface characterization and antibacterial activity of chitosan-grafted poly(ethylene terephthalate) prepared by plasma glow discharge. J. Appl. Polym. Sci..

[21] Chang Y.B., Tu P.C., Wu M.W., Hsueh T.H., Hsu S. (2008). A study on chitosan modification of polyester fabrics by atmospheric pressure plasma and its antibacterial effects. Fiber. Polym..

[22] Nigmatullin R., Konovalova V., Pobigay G. (2009). Development of Antimicrobial membranes via the surface tethering of chitosan. J. Appl. Polym. Sci..

[23] Tseng H.J., Hsu S., Wu M.W., Hsueh T.H., Tu P.C. (2009). Nylon textiles grafted with chitosan by open air plasma and their antimicrobial effect. Fiber. Polym..

[24] Abdou E.S., Elkholy S.S., Elsabee M.Z., Mohamed E. (2008). Improved antimicrobial activity of polypropylene and cotton nonwoven fabrics by surface treatment and modification with chitosan. J. Appl. Polym. Sci..

[25] Hu S.G., Jou C.H., Yang M.C. (2003). Antibacterial and biodegradable properties of polyhydroxyalkanoates grafted with chitosan and chitooligosaccharides via ozone treatment. J. Appl. Polym. Sci..

[26] Hu S.G., Jou C.H., Yang M.C. (2002). Surface grafting of polyester fiber with chitosan and the antibacterial activity of pathogenic bacteria. J. Appl. Polym. Sci..

[27] El-tahlawy K.F., El-bendary M.A., Elhendawy A.G., Hudson S.M. (2005). The antimicrobial activity of cotton fabrics treated with different crosslinking agents and chitosan. Carbohyd. Polym..

[28] Yang M.C., Lin W.C. (2002). The grafting of chitosan oligomer to polysulfone membrane via ozone-treatment and its effect on anti-bacterial activity. J. Polym. Res..

[29] Nakajima N., Ikada Y. (1995). Mechanism of amide formation by carbodiimide for bioconjugation in aqueous-media. Bioconjug. Chem..

[30] Carey F.A., Sundberg R.J. (2007). Advanced Organic Chemistry, Part B: Reactions and Synthesis.

[31] Lehocký M., Drnovská H., Lapčíková B., Barros-Timmons A.M., Trindade T., Zembala M., Lapčík L. (2003). Plasma surface modification of polyethylene. Colloid Surf. A.

[32] Novák I., Pollák V., Chodák I. (2006). Study of surface properties of polyolefins modified by corona discharge plasma. Plasma Process. Polym..

[33] Sharma P.K., Rao K.H. (2002). Analysis of different approaches for evaluation of surface energy of microbial cells by contact angle goniometry. Adv. Colloid Interface Sci..

[34] Janćzuk B., Białopiotrowicz T., Zdziennicka A. (1999). Some remarks on the components of the liquid surface free energy. J. Colloid Interface Sci..

[35] James N.R., Jayakrishnan J.A. (2003). Surface thiocyanation of plasticized poly (vinyl chloride) and its effect on bacterial adhesion. Biomaterials.

[36] Yang M.R., Chen K.S., Tsai J.C., Tseng C.C., Lin S.F. (2002). The antibacterial activities of hydrophilic-modified nonwoven PET. Mater. Sci. Eng..

[37] Chen K.S., Ku Y.A., Lin H.R., Yan T.R., Sheu D.C., Chen T.M. (2006). Surface grafting polymerization of N-vinyl-2-pyrrolidone onto a poly(ethylene terephthalate) nonwoven by plasma pretreatment and its antibacterial activities. J. Appl. Polym. Sci..

[38] Vesel A., Junkar I., Cvelbar U., Kovač J., Mozetič M. (2008). Surface modification of polyester by oxygen- and nitrogen-plasma treatment. Surf Interface Anal..

[39] Gupta B., Plummer C., Bisson I., Frey P., Hilborn J. (2002). Plasma-induced graft polymerization of acrylic acid onto poly (ethylene terephthalate) films: Characterization and human smooth muscle cell growth on grafted films. Biomaterials.

[40] Sartori S., Rechichi A., Vozzi G., D’Acunto M., Heine E., Giusti P., Ciardelli G. (2008). Surface modification of a synthetic polyurethane by plasma glow discharge: Preparation and characterization of bioactive monolayers. React. Func. Polym..

[41] Gupta B., Shalini S., Ray A. (2008). Plasma induced graft polymerization of acrylic acid onto polypropylene monofilament. J. Appl. Polym. Sci..

[42] Stuart B. (2004). In Infrared Spectroscopy: Fundamentals and Applications.

[43] Kumagai H., Tashiro T., Kobayashi T. (2005). Formation of conjugated carbon bonds on poly (vinyl chloride) films by microwave-discharge oxygen-plasma treatments. J. Appl. Polym. Sci..

[44] Chan C.M. (1994). In Polymer Surface Modification and Characterization.

[45] Balazs D.J., Triandafillu K., Chevolot Y., Aronsson B.O., Harms H., Descouts P., Mathieu H.J. (2003). Surface modification of PVC endotracheal tubes by oxygen glow discharge to reduce bacterial adhesion. Surf. Interface Anal..

[46] An Y.H., Friedman R.J. (1998). Concise review of mechanisms of bacterial adhesion to biomaterial surfaces. J. Biomed. Mater. Res..

[47] Tortora G.J., Funke B.R., Case C.L. (2006). In Microbiology:An Introduction.

[48] Zhang W., Chu P.K., Ji J., Zhang Y., Ng S.C., Yan Q. (2006). Surface antibacterial characteristics of plasma-modified polyethylene. Biopolymers.

